# Influence of the Substrate to the LSP Coupling Wavelength and Strength

**DOI:** 10.1186/s11671-018-2691-2

**Published:** 2018-09-10

**Authors:** Jiawei Liao, Li Ji, Jin Zhang, Na Gao, Penggang Li, Kai Huang, Edward T. Yu, Junyong Kang

**Affiliations:** 1 Fujian Provincial Key Laboratory of Semiconductors and Applications, Collaborative Innovation Center for Optoelectronic Semiconductors and Efficient Devices, Department of Physics, Xiamen University, Xiamen, 361005 People’s Republic of China; 2 Department of Electrical and Computer Engineering, Microelectronic Research Center, The University of Texas at Austin, Austin, TX 78758 USA; 3 Inspection and Quarantine Technology Center, Xiamen Entry-Exit Inspection and Quarantine Bureau of the People’s Republic of China, Xiamen, 361026 People’s Republic of China

**Keywords:** Localized surface plasmon, Dielectric interface, Resonance wavelength, Coupling strength

## Abstract

Three kinds of typical structures, hemi-/spherical nanoparticles/nanoparticle dimers on the substrate and spherical nanoparticles/nanoparticle dimers half-buried into the substrate, are used for FDTD simulation to theoretically discuss the influence of the substrate to the localized surface plasmon (LSP) coupling when the metal nanoparticles/nanoparticle dimers are locating near a substrate. Simulated results show that the dependencies between the LSP coupling wavelength and the refractive index of the substrate for different structures are not the same, which can be attributed to the different polarization field distributions of LSPs. When light is incident from different directions, the LSP coupling strength are not the same as well and the ratios of the scattering peak intensities depend on the position of the metal nanoparticles or nanoparticle dimers. These phenomenon can be explained by the difference of the local driving electric field intensities which is modulated by the interface between the air and the substrate.

## Background

Localized surface plasmon (LSP) is a strong coupling phenomena between electrons in noble metal nanoparticles (NPs) and incident light when the size of NPs is comparable to or smaller than the wavelength of incident light. The LSP resonance wavelength depends on the size, shape, and material of NPs as well as the surrounding dielectric environment [1–4]. Because of its many attractive features, including exponentially enhanced electric fields near the interface between metal and dielectric medium and enhanced absorption at the plasmon resonant wavelength [5, 6], LSPs have been integrated into many optoelectronic devices, including light-emitting diodes (LEDs) [7–9], photodetectors [10, 11], solar cells [12, 13], and other emerging technologies such as surface-enhanced Raman scattering (SERS) [14–17], tip-enhanced Raman scattering (TERS) [18, 19], and chemical sensors [20, 21].

For most of LSP-based applications, substrates that support the metal NPs is inevitable. In previous studies, studies for the influence of substrates are usually focused on the refractive index of substrates or the separation between particles and substrates [22, 23]. Particularly for metal nanoparticles with cubic geometry, substrates will induce the hybridization between dipolar and quadrupolar cube modes [24, 25]. The influence of substrates is neglected by using an effective refractive index theory. However, in our previous work, we have discussed the different LSP coupling strengths when light is incident from different directions when hemispherical metal NPs are located on a substrate, which can be attributed to the different localized electric field intensities originating from Fresnel reflection of the interface [26]. In this work, three structures with Au NPs located on substrate are used for FDTD simulation to discuss the coupling wavelengths and strength of the LSPs. The first structure is hemispherical metal NPs on a substrate, which can be obtained by physical methods such as thermal annealing or nanoimprint [27–29]. The second structure is spherical metal NPs on substrate, which is usually obtained by chemical synthesis and subsequent transferring process [30, 31]. These two structures are typically utilized for a solid substrate. The third structure is spherical metal NPs half buried into the substrate, which have been observed on a liquid-liquid interface [32]. Our results show that for different structures, the effective refractive index of the medium surrounding the NPs behaves differently. The coupling wavelengths of the first and the third structures redshift greatly with the increase of the refractive indices of the substrate while the coupling wavelength of the second structure remains almost constantly. This can be attributed to various degrees of penetration into the substrate of the polarization electric field. In addition, the LSP coupling strengths of these three structures have also been studied by tuning the direction of incident light, normally either from air or substrate. Simulated results show that for the first and second structures, when light is incident from different directions, the ratio of the scattering peak intensities is equal to the ratio of the refractive indices of the incidence medium and the exiting medium. However, for the third structure, these two ratios do not equal to each other. These behaviors can be quantitatively explained by considering the local driving electric field intensities of the LSPs using modified Fresnel equations.

However, in the practice, array structure of nanoparticles is usually achieved for investigation. Thus NP dimers [33–35] have also been employed for discussion because the near field properties of the periodic NP structures will be affected by boundary condition issues in FDTD simulations. The FDTD simulation results demonstrate that trends of the coupling wavelengths and strengths of the metal NP dimers are mostly similar to that of the single metal NP for the first and third structures. However, for metal NP dimers with the second structure, the influence of the refractive index of the substrate is slightly stronger than that for the single metal NP.

## Result and Discussion

**Fig. 1:**
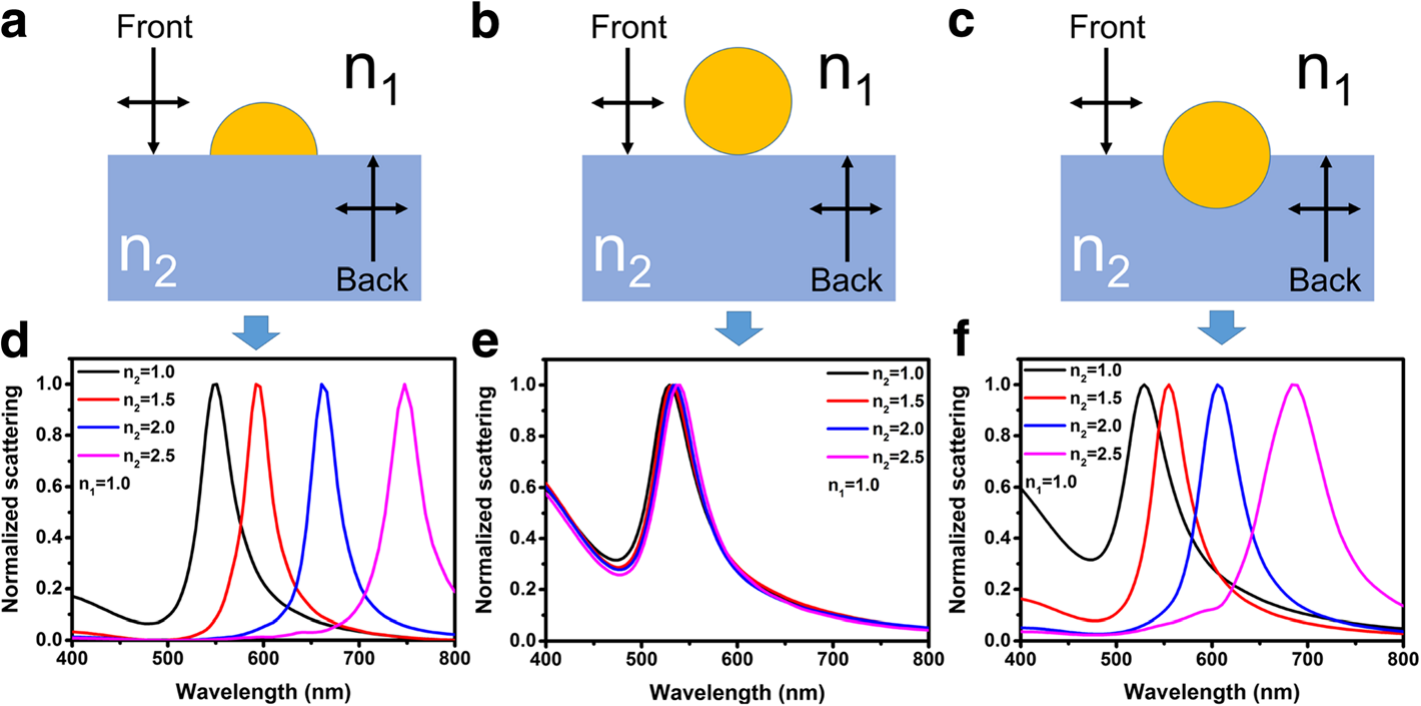
**a**–**c** Schematic diagrams of structure A to C used for FDTD simulations respectively. **d**–**f** Normalized scattering spectra of structure A to C with varying substrate refractive indices respectively

Figure 1a–c shows the schematic illustrations of the structures for FDTD simulations. The structure shown in Fig. 1a represents the semispherical Au NPs on a dielectric substrate, which is named as structure A. The structures shown in Fig. 1b represent the spherical Au NPs on a dielectric substrate which is named as structure B. For comparison, the structure C shown in Fig. 1c, which have a higher symmetry, is used for simulations as well. For simulation, diameters of the Au NPs for all structures are set as 60 nm. The refractive indices of the mediums above the substrates are set as *n*_1_ = 1 in most cases. The refractive indices of the substrates vary from *n*_2_ = 1 to *n*_2_ = 2.5. Figure 1d–f shows the normalized scattering spectra of structures A to C, respectively. It is clearly to see that for structure A and C, the scattering peaks redshift with the increasing of the refractive indices of the substrates dramatically. However, for structure B, the increase of the refractive indices of the substrates has a negligible effect on the scattering peaks.

**Fig. 2:**
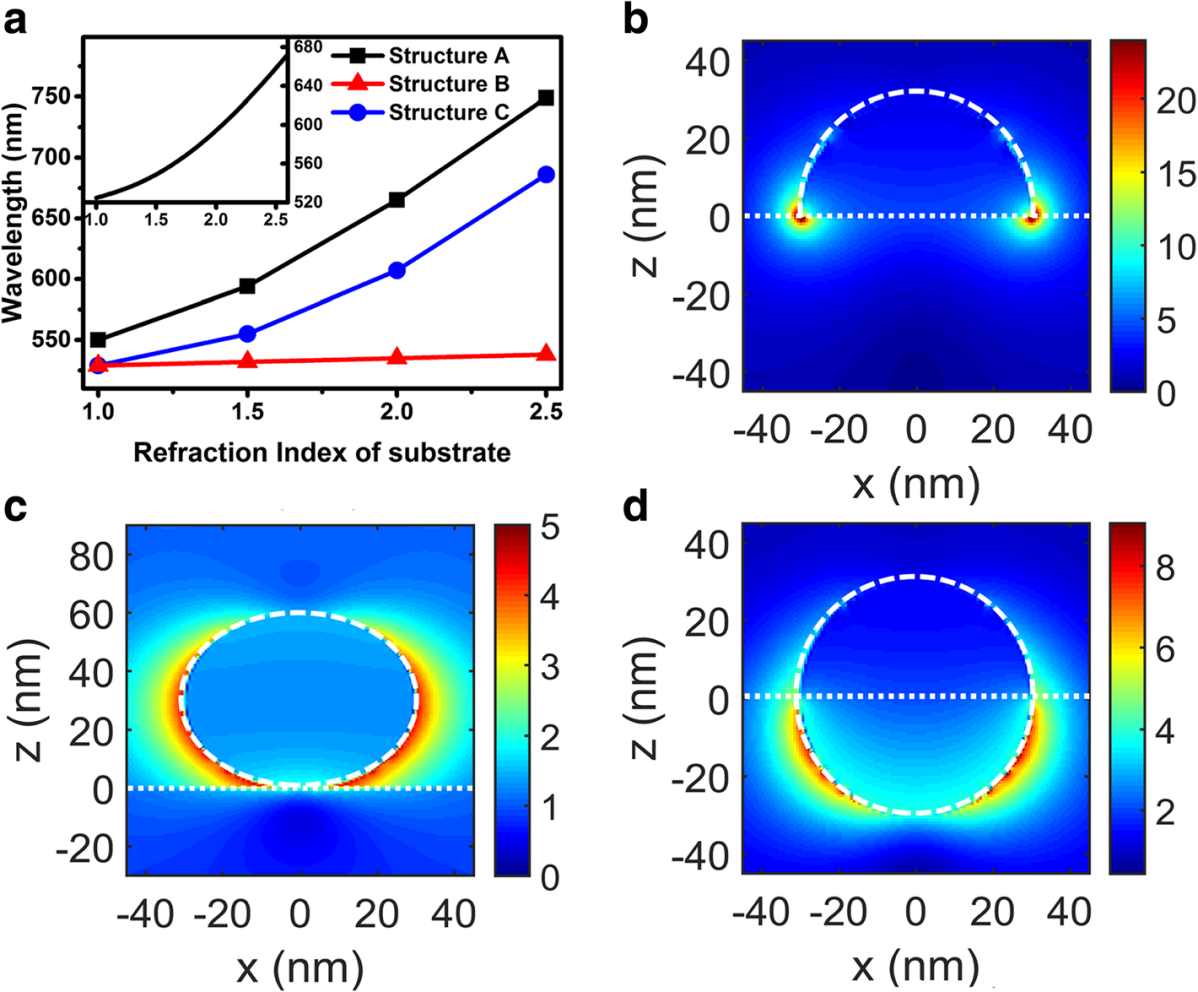
**a** Scattering peak wavelengths of different structures with varying substrate refractive indices. The insert shows the relationship between the LSP coupling wavelength and refractive index of surrounding medium based on Mie theory. **b**–**d** Polarization electric field distributions of structure A to C with *n*_2_ = 1.5 at the corresponding LSP coupling wavelength respectively

Figure 2a shows the wavelength of the LSP scattering maxima vs. the refractive indices of the substrates extracted from Fig. 1. From Fig. 2a, the first information we can obtain is that when the refractive indices of substrates increase, the scattering peak wavelengths increase faster than the linearly assumption. This can be approximately explained by the Mie theory. From Mie theory, under the Quasi-Static Approximation, the scattering cross section of a metal NP surrounded by an isotropic and non-absorbing medium with dielectric constant *ε*_*m*_ can be expressed as:

1 $$ {C}_S=\frac{8\pi }{3}{k}^4{a}^6{\left|\frac{\varepsilon -{\varepsilon}_m}{\varepsilon +2{\varepsilon}_m}\right|}^2 $$

where *k* is the wave vector of the propagating wave, *a* is the radius of a spherical metal NP, and *ε* represents the dielectric constant of the metal. Insert in the Fig. 2a shows the relationship between the scattering peak wavelengths and the refractive indices of the medium surrounding the metal NP calculated using Eq. (1). One can clearly see the super-linear relationship between the scattering peak wavelengths and the refractive indices which is quite similar to the simulated results. Thus we can use the effective refractive index theory for further discussions. From the effective refractive index theory, if the scattering peak wavelengths of Au NP are surrounded by an infinite dielectric medium with refractive index *n*_*eff*_ equal to that of the Au NP for different structures, *n*_*eff*_ can be regarded as the effective refractive indices of the corresponding structures. Table 1 shows the *n*_*eff*_ obtained using this method.

**Table 1:** *n*_eff_ for different structures and with varying substrate refractive indices

Structure	*n*_2_	1.00	1.50	2.00	2.50
	*n*_eff_				
Structure A		1.00	1.30	1.63	1.96
Structure B		1.00	1.03	1.08	1.12
Structure C		1.00	1.35	1.75	2.185

Using a linear fitting equation [36]:

2 $$ {n}_{eff}=\mu {n}_1+\left(1-\mu \right){n}_2, $$

where *μ* can be regarded as the weighting coefficient to estimate the influence of substrate refractive index to the LSP coupling wavelength. The influence of the mediums above and below the interface can be estimated. Using the parameters shown in Table 1, the weighting coefficients *μ* of structure A to C are 0.38 ± 0.02, 0.93 ± 0.01, and 0.25 ± 0.05, respectively. These results indicate that for structure B, the scattering peak wavelength is almost dependent on the refractive index of the medium above the interface only. For structure C, the refractive index of the substrate plays an important role to the scattering peak wavelength. However, for structure A, the scattering peak wavelength is affected by the refractive index of the mediums above and below the interface both.

These phenomena can be explained by the electric field distributions analysis. Figure 2b–d shows the electric field amplitude distributions of structure A to C with *n*_2_ = 1.5 at the corresponding scattering peak wavelengths respectively. Electric field concentrated mostly near the interface, both the medium above the interface and the medium below the interface affect the resonance wavelengths of the LSPs for structure A to C, respectively. These results confirm that the electric field distribution is in good agreement with the calculated weighting coefficients because the influence of the surrounding medium to the scattering peak wavelength can be attributed to the polarization of the dielectric medium caused by the localized electric field.

**Fig. 3:**
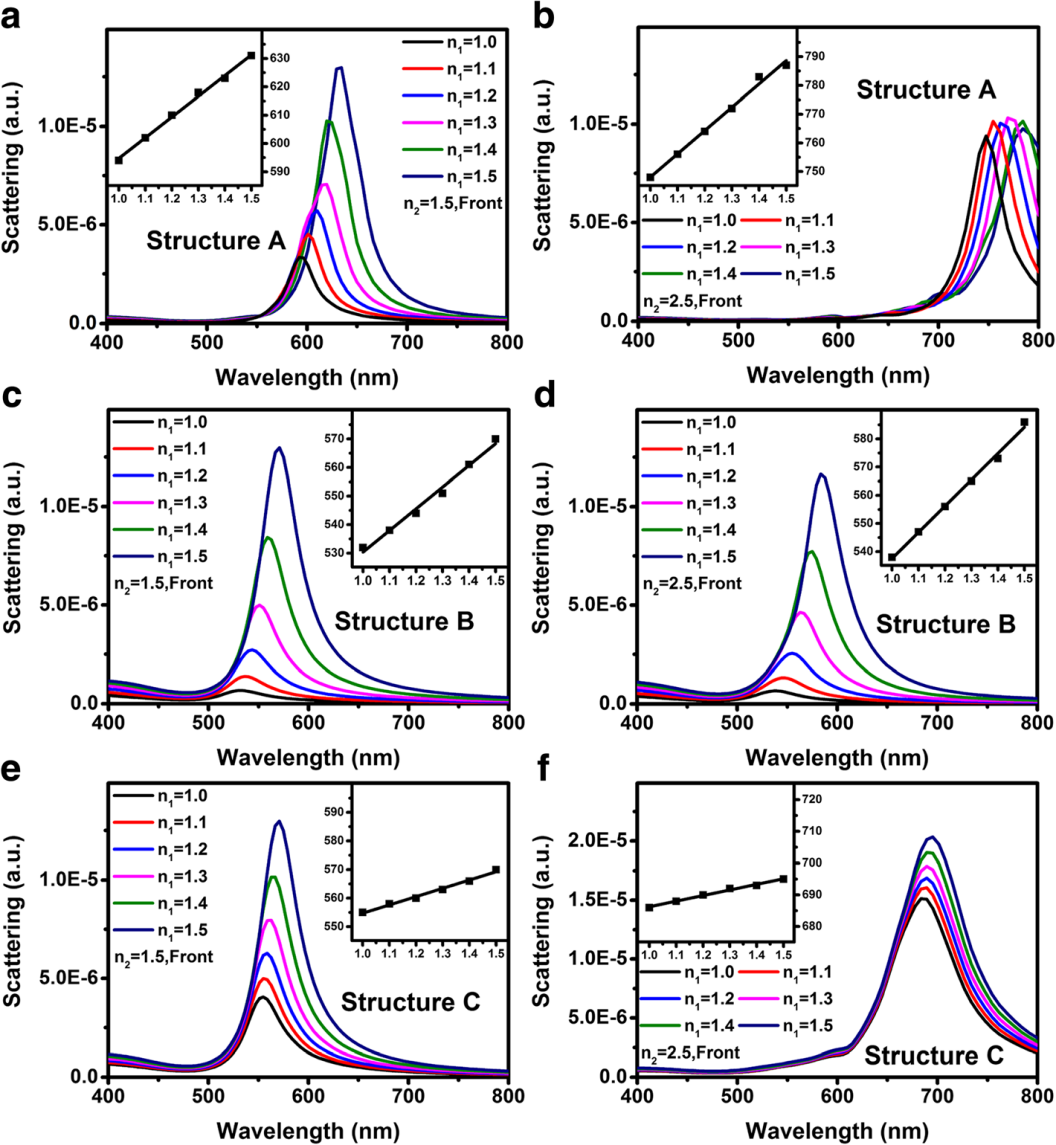
**a**, **c**, **e** Scattering spectra of structure A to C when *n*_1_ is linearly increased from 1.0 to 1.5. with fixed *n*_2_ = 1.5, respectively. **b**, **d**, **f** Scattering spectra of structure A to C when *n*_1_ is linearly increased from 1.0 to 1.5, with fixed *n*_2_ = 2.5, respectively. The inserts show the relationship between scattering peak wavelengths and *n*_1_ for different structures or substrate refractive indices

From Eq. (2), we obtain when *n*_2_ is fixed and *n*_1_ is tunable, the changing rate, i.e., the slope of the *n*_*eff*_, is the weighting coefficients *μ*. Thus we can use the results above to optimize the LSP-based chemical sensor if the substrate is unavoidable. LSP-based chemical sensor is to detect the refractive index changing of surrounding environment through the LSP resonance peak wavelength shift Δ*λ* [37]. The sensitivity of the sensors is strongly related to two parameters, including the shift parameter *S* = *d*(Δ*λ*)/*d*(Δ*n*) and the figure of merit *FOM* = *S*/*FWHM*, where Δ*n* represents the change of refractive index and *FWHM* is the full wave at half maximum of initial state [37, 38]. Most of previous studies on LSP-based sensors focus on the material, size, and the shape of the NPs [39–41]. However, very few reports discussed the influence of substrate and their interactions with the metal NPs. Figure 3 shows the scattering spectra of structure A to C when *n*_1_ is linearly increased from 1.0 to 1.5 and *n*_2_ is fixed as 1.5 or 2.5. Inserts shown in all figures represent the scattering peak wavelengths vs. *n*_1_. Figure 3a–f shows that the *S* parameter for structure A and B is higher than that of structure C. Table 2 lists the calculated parameters of *S*, *FWHM*, and *FOM* from Fig. 3. For *n*_2_ = 1.5, the *S* and *FOM* parameters for structures A and B is much better than that of structure C. However, for *n*_2_ = 2.5, although the *S* parameters for structures A and B is higher than that when *n*_2_ = 1.5, the *FOM* deteriorates because of the increasing of *FWHM*.

**Table 2:** Scattering peak wavelengths, performance parameters *S* and *FOM* of structure A to C calculated from Fig. 3

Model	*n*_2_	*S* (nm/RIU)	*FWHM* (nm)	*FOM* (RIU^−1^)
Structure A	1.5	74	46	1.61
	2.5	78	56	1.36
Structure B	1.5	76	44	1.73
	2.5	96	64	1.50
Structure C	1.5	30	52	0.54
	2.5	18	74	0.24

**Fig. 4:**
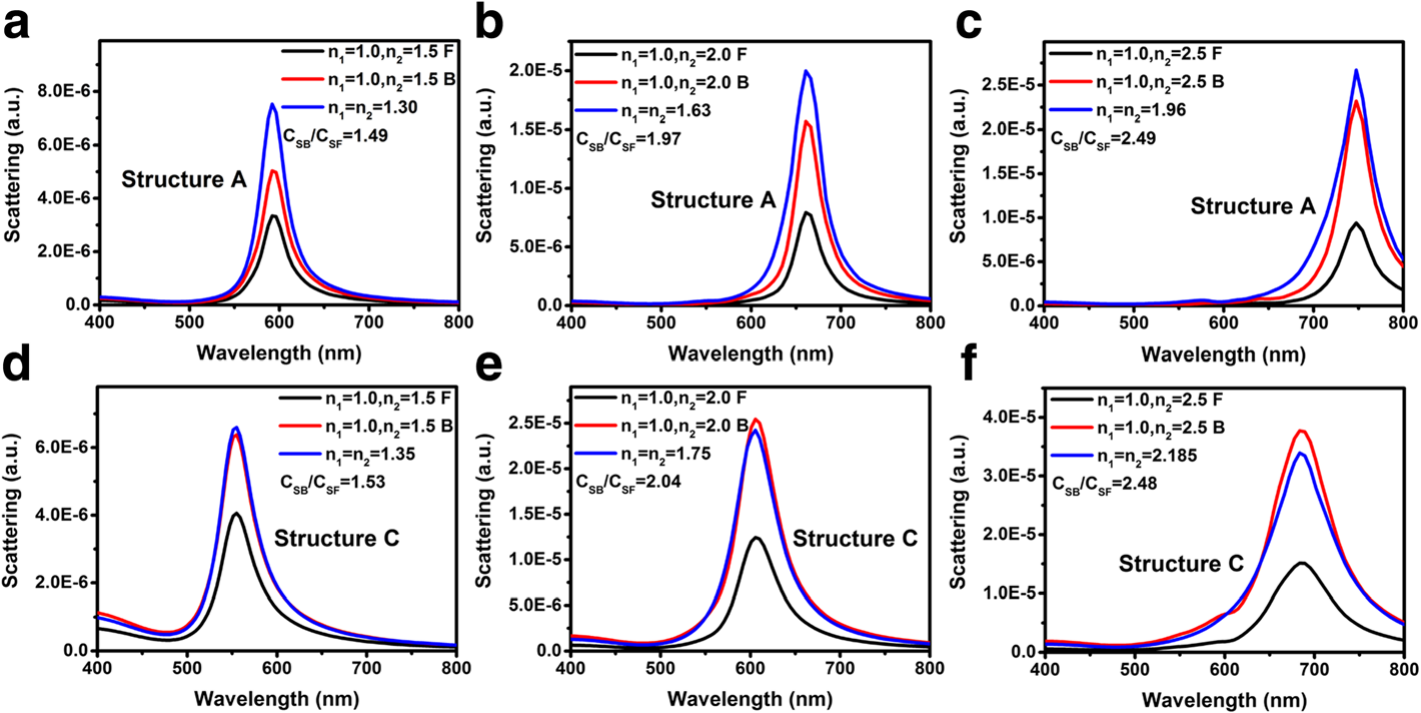
Scattering spectra for varying *n*_2_ = 1.5, 2.0, and 2.5 of structure A (**a**–**c**) and structure C (**d**–**f**) respectively. Light is incident normally from air (denoted as black lines) and substrates (denoted as red lines). The blue lines show the scattering spectra of which the Au NPs are surrounded by infinite dielectric mediums with effective refractive indices

The discussion above is all about the LSP coupling wavelength. While, the LSP coupling strength is another valuable parameter for many LSP-based devices such as LEDs, photodetectors, solar cells, and emerging techniques such as SERS, TERS, and chemical sensors. Our previous investigation indicated that for structure A, the coupling strength between light and LSPs will be influenced by the incident direction of light. This can be attributed to the different local driving electric field intensities when light is normally incident from the air and the substrate [26]. The ratio of the extinction peak intensities when light is incident from the substrate (denoted as back incidence) and the air (denoted as front incidence) *C*_*B*_/*C*_*F*_ is equal to *n*_2_/*n*_1_. Figure 4 shows the FDTD-simulated scattering spectra when light is incident from different directions, associated with the scattering spectra of Au NPs surrounded by the corresponding effective refractive indices. Figure 4a–c, d–f represents the scattering spectra of structures A and C respectively. The refractive indices of the substrate *n*_2_ are 1.5, 2.0, and 2.5 for Fig. 4a, d, b, e, c, f, respectively. *n*_1_ is fixed as 1.0 for all spectra. Similar to the extinction spectra, the scattering peak intensities when light is incident from back and front *C*_*SB*_/*C*_*SF*_ is equal to *n*_2_/*n*_1_ for structure A and C both.

**Fig. 5:**
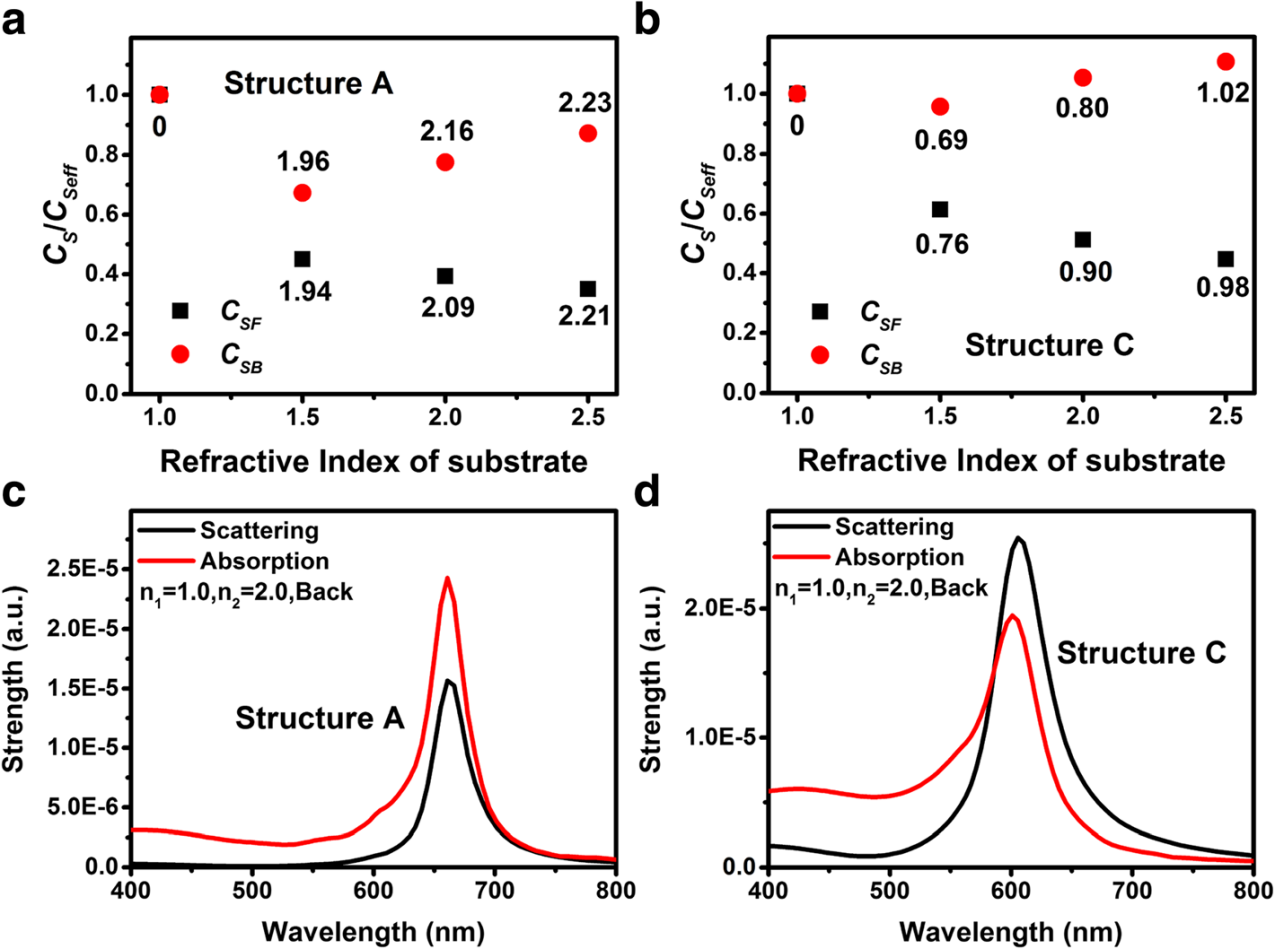
**a**, **b** The ratios of coupling strengths *C*_*S*_*/C*_*Seff*_ with various *n*_2_ of structure A and structure C respectively. Black rectangular and red circular dots represent the front and back incident cases respectively. **c**, **d** Scattering and absorption spectra of structures A and C with fixed *n*_2_ = 2.0 when light is incident from the substrate

When we take the scattering spectra of Au NPs surrounded by the corresponding effective refractive indices into account, there are difference between the scattering peak intensities of structure A and C. Figure 5a, b shows the ratios of *C*_*SF*_/*C*_*Seff*_ and *C*_*SB*_/*C*_*Seff*_ vs. the refractive indices of substrates of structure A and C respectively, where *C*_*Seff*_ is the scattering peak intensities of which the Au NPs are surrounded by infinite dielectric mediums with effective refractive indices (Fig. 4). For all substrates, the ratios *C*_*SF*_/*C*_*Seff*_ and *C*_*SB*_/*C*_*Seff*_ of structure A are smaller than those of structure C. This can also be explained by the difference between the local driving electric field of structure A and C.

Based on the modified Fresnel equations [26, 42], the intensity of the local driving electric field when light is incident from the front and back sides can be written as 2*n*_1_*E*_*i*_/(*n*_1_ + *n*_2_ + *A*) and 2*n*_2_*E*_*i*_/(*n*_1_ + *n*_2_ + *A*), where *E*_*i*_ is the electric field intensity of the incident wave, and *A* =  − *i*(*ω*/*c*)*ρα* can be regarded as an additional parameter arising from the LSPs, which is proportional to the polarizability *α* of the Au NPs and is a positive real number at the LSP resonance frequency. Thus the *C*_*SB*_/*C*_*SF*_ is equal to *n*_2_/*n*_1_ which is shown in Fig. 4 as well. On the other hand, the local driving electric field intensity when the Au NPs surrounded by the corresponding effective refractive index is equal to *E*_*i*_. Thus the value of *A* parameter can be obtained using the equation:

3 $$ \frac{2{n}_1}{n_1+{n}_2+A}=\frac{C_{SF}}{C_{Seff}},\mathrm{and}\ \frac{2{n}_2}{n_1+{n}_2+A}=\frac{C_{SB}}{C_{Seff}}. $$

The calculated *A* parameters are listed near the corresponding point in Fig. 5a, b. One can see that the value of *A* is very close but not exactly the same for different light incident directions. This is attributed to the slightly difference between *C*_*SB*_/*C*_*SF*_ and *n*_2_/*n*_1_ as well as the accuracy of the simulation software. For the same structure with different substrate refractive indices, the *A* value increases with the increasing of the substrate refractive indices, which can be attributed to the increased polarizability of the Au NPs with the increasing of the LSP resonance wavelength [43–45]. One the other hand, one should be aware that the *A* value of structure A is much bigger than that of structure C for different structures with the same substrate refractive indices. It means that the polarizability of the Au NPs for structure A is much bigger than that of structure C, which can be proven by Fig. 2b, d. It is interesting that although the polarizability of the Au NPs of structure A is bigger than that of structure C, the scattering peak intensities of structure A is smaller than that of structure C (Fig. 4). This can be attributed to the higher absorption of structure A. Figure 5c, d shows the scattering and absorption spectra of structure A and C respectively, the refractive index of the substrate is 2.0 for both structures and light is incident from back side. One can see that the absorption of structure A is much higher than that of structure C. Thus for structure A, most of the energy that excite the LSPs is consumed via absorption and does not scattered.

**Fig. 6:**
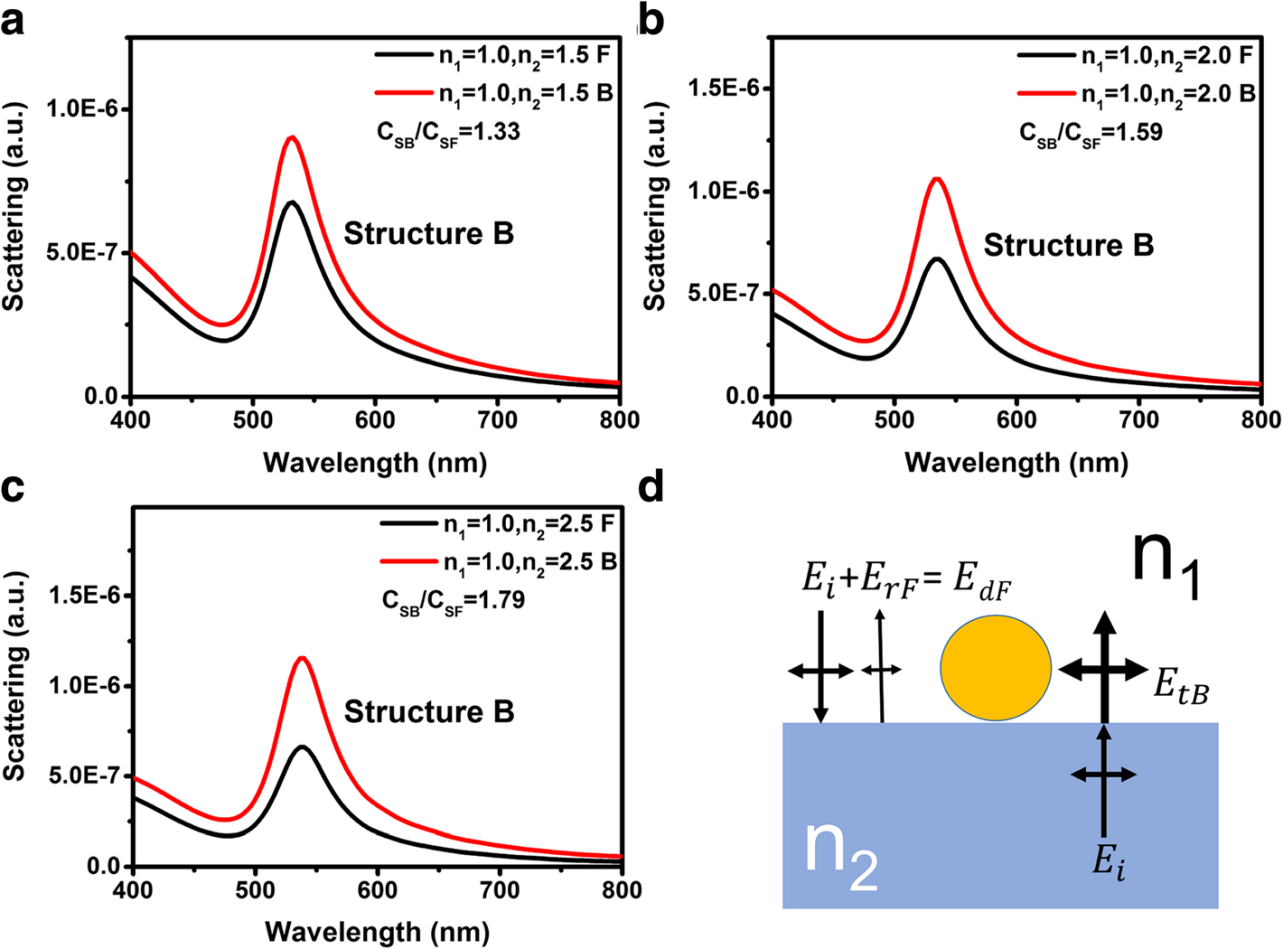
**a**–**c** Scattering spectra for varying *n*_2_ = 1.5, 2.0, and 2.5 of structure B respectively. Black and red lines represent the front and back incident cases respectively. **d** Schematic diagram of local driving electric field of structure B for different incident directions

However, for structure B, the ratio *C*_*SB*_/*C*_*SF*_ does not equal to *n*_2_/*n*_1_. Figure 6a–c presents the scattering spectra of structure B with different substrate refractive indices of 1.5, 2.0, and 2.5 respectively. *C*_*SB*_/*C*_*SF*_ of structure B is smaller than *n*_2_/*n*_1_ for all substrate refractive indices. As schematically illustrated in Fig. 6d, when light is incident from front side, the local driving electric field can be written as the superposition of *E*_*i*_ and *E*_*rF*_, where *E*_*rF*_ is the electric field intensity of the reflected wave. The local driving electric field intensity when light is incident from the front side can be written as ${E}_{dF}={E}_i+{E}_{rF}=\left[1+\frac{n_1-{n}_2}{n{}_1+{n}_2}\cos \left(\frac{4\pi Pa}{\lambda_{LSP}}\right)\right]{E}_i$, where *P* is a coefficient that relate to the average distance of the oscillating electrons and an additional light path when light is propagating through the Au NPs, and the *λ*_*LSP*_ is the resonance wavelength of the LSPs. Considering that the local driving electric field intensity when light is incident from the back side can be written as *E*_*dB*_ = *E*_*tB*_ = 2*n*_2_*E*_*i*_/(*n*_1_ + *n*_2_), the ratio of the local driving electric field intensities when light is incident from back and front sides can be written as:

4 $$ \frac{E_{dB}}{E_{dF}}=\frac{2{n}_2}{\left(n{}_1+{n}_2\right)+\left({n}_1-{n}_2\right)\cos \left(4\pi Pa/{\lambda}_{LSP}\right)} $$

Table 3 listed the *C*_*SB*_/*C*_*SF*_ of structure B obtained by the scattering spectra and the *E*_*dB*_/*E*_*dF*_ calculated using Eq. (4) with different *P* coefficients. One can see that when the *P* coefficient equals to 1.5, *E*_*dB*_/*E*_*dF*_ is in good accord with the ratios of *C*_*SB*_/*C*_*SF*_ for all substrates. The reason why *P* equals to 1.5 is still unclear.

**Table 3:** Simulated *C*_*SB*_/*C*_*SF*_ and calculated *E*_*dB*_/*E*_*dF*_ using Eq. (4) of structure B with different *P* coefficients

*n*_2_	Peak wavelengths (nm)	*C*_*SB*_/*C*_*SF*_	*E*_*dB*_/*E*_*dF*_		
			*P* = 1.0	*P* = 1.5	*P* = 2.0
1.5	532	1.33	1.41	1.33	1.24
2.0	535	1.59	1.79	1.59	1.41
2.5	538	1.79	2.12	1.81	1.54

Tables 4 and 5 listed the *C*_*SB*_/*C*_*SF*_ obtained by the scattering spectra and the *E*_*dB*_/*E*_*dF*_ calculated using Eq. (4) for NPs with different geometric structures and materials to investigate the universality of the *P* coefficient. One can see that for Au NPs with difference sizes, when the *P* coefficient is equal to 1.5, the ratios of *C*_*SB*_/*C*_*SF*_ and *E*_*dB*_/*E*_*dF*_ agree with each other quite well whenever the NPs are oblate elliptical or prolate elliptical. Table 5 shows that the *P* coefficient of the Ag NPs with different sizes equals to 1.5 as well. Thus the *P* coefficient is relatively universal, indicating there should be an internal mechanism for the *P* coefficient and worth further in-depth investigation.

**Table 4:** Simulated *C*_*SB*_/*C*_*SF*_ and calculated *E*_*dB*_/*E*_*dF*_ using Eq. (4) when *P* equals 1.5 of different sizes, shapes of Au NPs when *n*_2_ fixed as 1.5

Model	Axial length (nm)			*C*_*SB*_/*C*_*SF*_	*E*_*dB*_/*E*_*dF*_ (*P* = 1.5)
	*x*	*y*	*z*		
Au oblate ellipsoid	80	80	60	1.32	1.34
	75	75	50	1.36	1.38
	60	60	40	1.42	1.42
Au prolate ellipsoid	40	40	60	1.33	1.33
	33.4	33.4	50	1.38	1.37
	26.7	26.7	40	1.44	1.41

**Table 5:** Simulated *C*_*SB*_/*C*_*SF*_ and calculated *E*_*dB*_/*E*_*dF*_ using Eq. (4) when *P* equals 1.5 of different sizes and material of spherical NPs when *n*_2_ fixed as 1.5

Model	Axial length (nm)			*C*_*SB*_/*C*_*SF*_	*E*_*dB*_/*E*_*dF*_ (*P* = 1.5)
	*x*	*y*	*z*		
Au sphere	60	60	60	1.33	1.33
	50	50	50	1.39	1.37
	40	40	40	1.43	1.41
Ag sphere	60	60	60	1.23	1.22
	50	50	50	1.30	1.28
	40	40	40	1.38	1.35

The discussions above are based on single NP. However, in the practice, array structures of nanoparticles are usually achieved for investigation. Thus NP dimers should be employed for discussion because the near field properties of the periodic NP structures will be affected by boundary condition issues in FDTD simulations. The geometric structure parameters of the NPs used for the dimer simulation are similar to that for the single NP discussed above, and a 2 nm gap is set between these two NPs. The simulated results (not shown here) demonstrated that when the polarization direction of normally incident light is perpendicular to the NP dimer, all properties is the same to that as shown for single NP. Thus all near field properties discussed below are based on an incident light of which the polarization direction is parallel to the NP dimer.

**Fig. 7:**
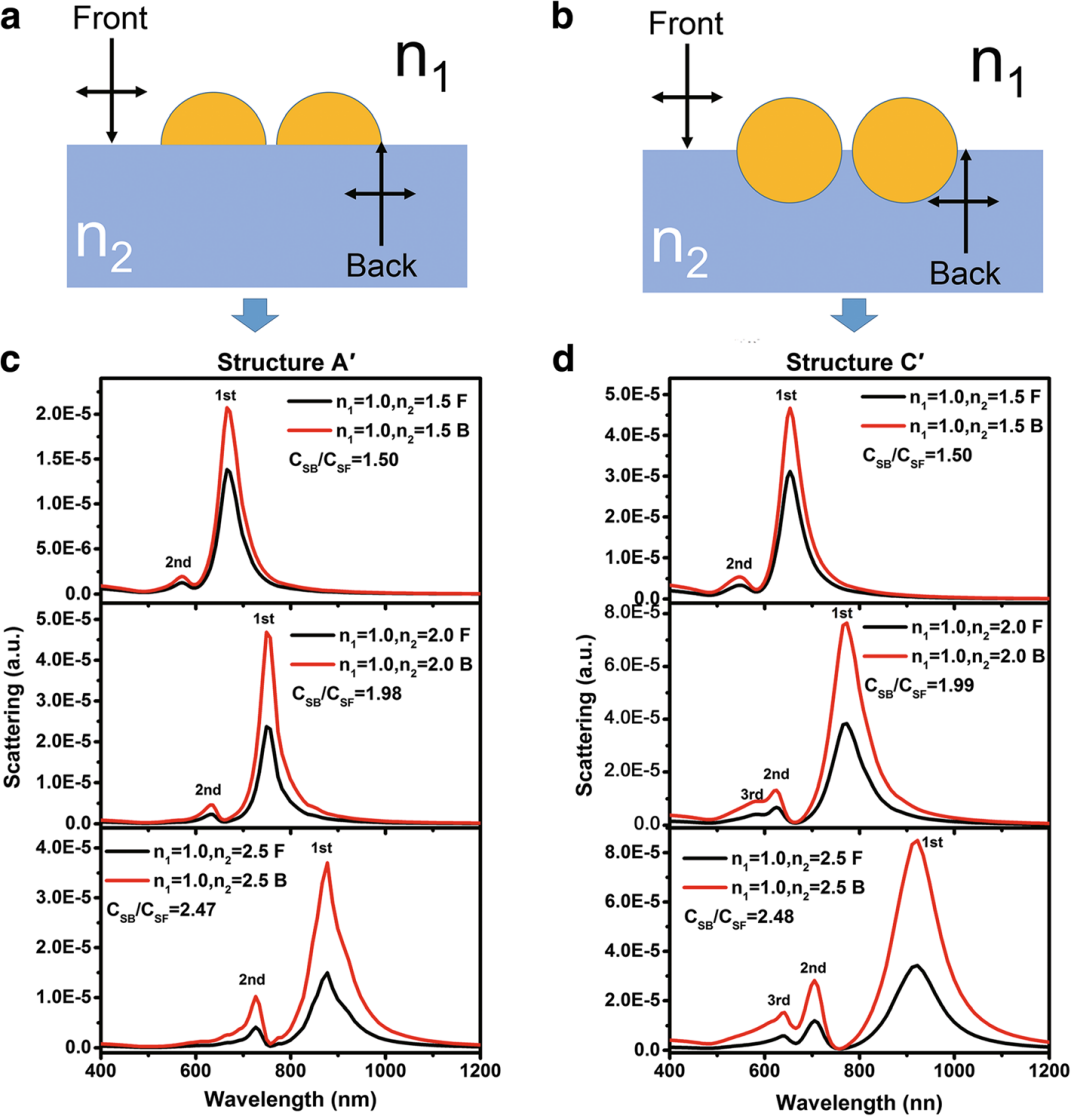
**a**, **b** Schematic diagrams of structure A′ and C′ used for FDTD simulations respectively. **c**, **d** Scattering spectra for varying *n*_2_ = 1.5, 2.0, and 2.5 of structure A′ and structure C′ respectively. Light is incident normally from air (denoted as black lines) and substrates (denoted as red lines)

**Fig. 8:**
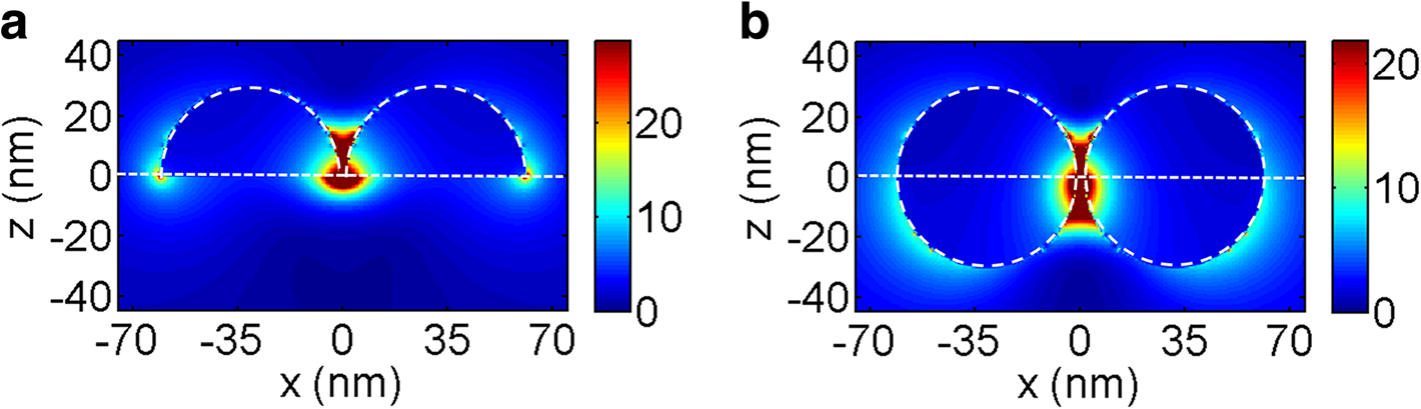
**a**, **b** Polarization electric field distributions of structure A′ and C′ with *n*_2_ = 1.5 at the corresponding wavelengths of the first order peak respectively

Figure 7a, b shows the schematic illustrations of semispherical Au dimers on a dielectric substrate (structure A′) and spherical Au dimers half-buried into the substrate (structure C′) respectively. Figure 7c, d shows the scattering spectra of the dimers with different substrate refractive indices and light incident directions. One can see that for structure A′ and C′, both the first-order and the second-order scattering peaks are observed in all spectra. Particularly, for structure C′, the third-order peaks can be observed when the refractive indices of the substrate is equal to 2 and 2.5. One can also see that all scattering peaks redshift greatly with the increase of the substrate refractive indices. This can be explained by the electric field amplitude distributions at the corresponding wavelengths of the first-order peak for structure A′ and C′ as shown in Fig. 8a, b, respectively, the refractive indices of the substrate is 1.5. Similar to that as shown in Fig. 2, the electric field concentrated mostly near the interface. Thus when light is incident from different directions, an equal of *C*_*SB*_/*C*_*SF*_ to *n*_2_/*n*_1_ can be expected and as demonstrated in Fig. 7c, d. On the other hand, comparing with the scattering spectra as shown in Fig. 4, the scattering peak intensities of the dimer are much higher than that of the single NP. This is attributed to the great electric field enhancement by the *hot spots* at the nano gaps [33].

**Fig. 9:**
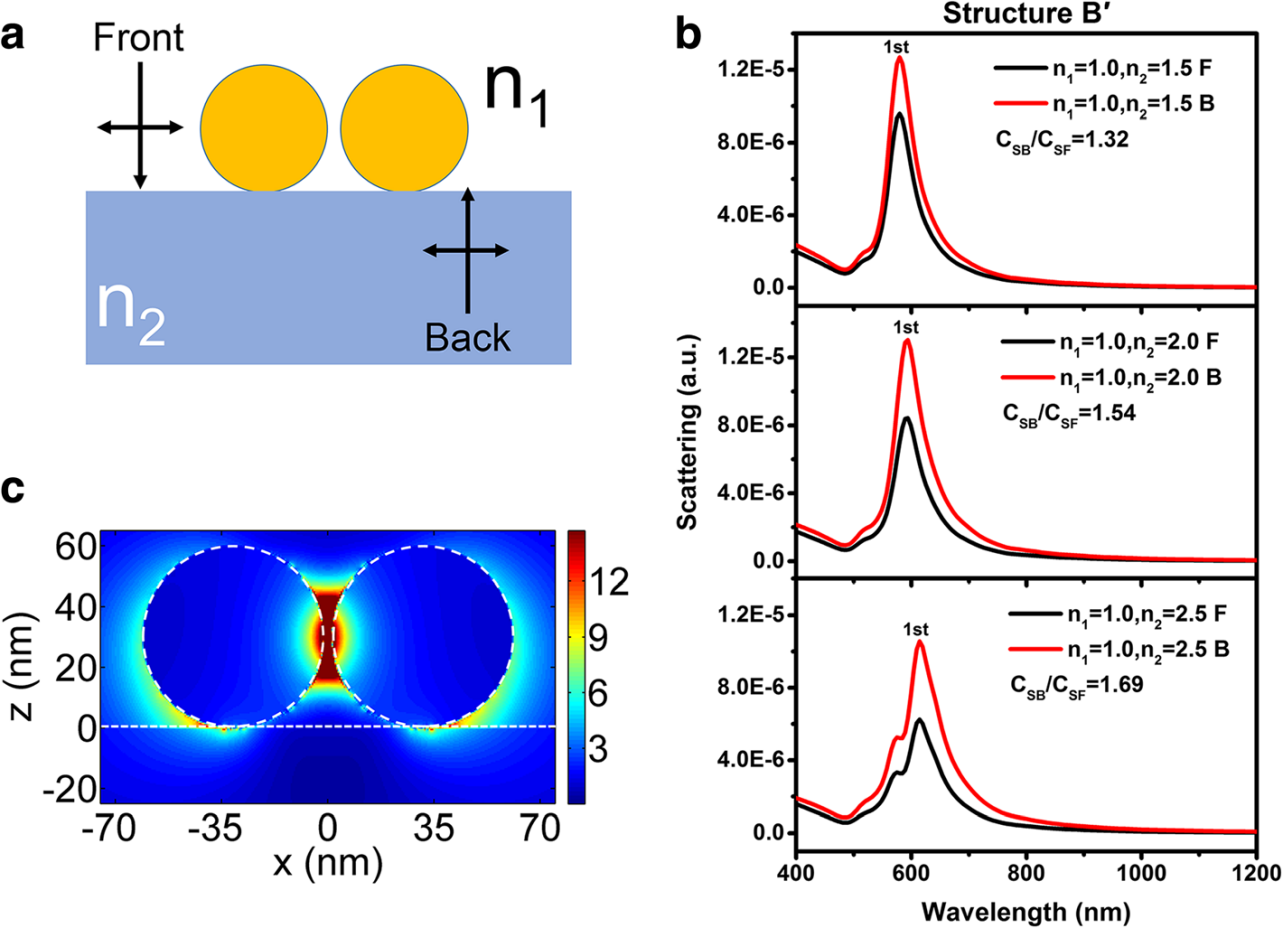
**a** Schematic diagrams of structure B′ used for FDTD simulations. **b** Scattering spectra for varying *n*_2_ = 1.5, 2.0, and 2.5 of structure B′. Light is incident normally from air (denoted as black lines) and substrates (denoted as red lines). **c** Polarization electric field distributions of structure B′ with *n*_2_ = 1.5 at 532 nm

However, as shown in Fig. 9, for spherical Au dimers located on a dielectric substrate (structure B′), the influence by the refractive index of the substrate is slightly stronger than that for structure B. The first-order peak redshifts from 580 to 614 nm when the refractive index of the substrate is increased from 1.5 to 2.5, of which is larger than that for single NP (from 532 to 538 nm). This may be attributed to the electric field amplitude distributions at the corresponding peak wavelength of the first-order peak for structure B′ (Fig. 9d, the refractive index of the substrate is 1.5). The electric field intensity in the substrate is stronger than that shown in Fig. 2c. As well, as shown in Fig. 9, the ratio of *C*_*SB*_/*C*_*SF*_ for NP dimers of structure B′ does not equal to *n*_2_/*n*_1_, similar to that for single NP. However, the *P* parameter is no longer a constant if Eq. (4) is still applied. The *P* parameters can be calculated to 1.67, 1.82, and 2.05 when the refractive index of the substrate is 1.5, 2.0, and 2.5, respectively. The difference between the *P* parameter for structure B and B′ needs further investigations.

## Conclusions

In summary, the impact of the substrate on the coupling wavelength and strength of LSPs have been studied by FDTD simulation and theoretical analysis. For the structures with hemispherical Au NPs located on substrate and spherical Au NPs half-buried into the substrate, the LSP coupling wavelength varies greatly with the refractive index of the substrate. However, the dependency of the LSP coupling wavelength onto substrate is marginal for the structure that spherical Au NPs are located on the substrate. The dependency difference has been explained by the polarization field distributions of LSPs for different structures. For the structure of which spherical Au NPs is half-buried into the substrate, the polarization field of LSPs is concentrated in the medium above the substrate. However, the polarization fields penetrate into the substrate greatly for the other two structures. In addition, the LSP coupling strengths of these three structures have also been studied by changing the incident direction of light, either normally from air or substrate. Simulated results show that for the structures with hemispherical NPs located on the substrate and spherical NPs half-buried into the substrate, the ratio of the scattering peak intensities for different light incident directions is equal to the ratio of the refractive indices of the incidence medium and the exiting medium. However, for the structure of which spherical NPs are located on the substrate, these two ratios do not equal to each other. These phenomena have been quantitatively explained by considering the local driving electric field intensities of the LSPs using modified Fresnel equations. The near field property of NP dimers is also calculated. Although multiple order peaks are shown in the scattering spectra, the scattering peak wavelengths redshift greatly for structures with substrate refractive indices for hemispherical Au dimers located on substrate and spherical Au dimers half-buried into the substrate. The ratio of the scattering peak intensities for different light incident directions is equal to the ratio of the refractive indices of the incidence medium and the exiting medium as well. However, for Au dimers located on the substrate, the influence induced by the refractive index of the substrates is slightly stronger than that for single spherical Au NP located on the substrate.

## Methods

The models of hemi-/spherical metal NP located on substrate (denoted as structures A and B) and spherical metal NP half-buried into substrate (denoted as structure C) are created and studied by Lumerical FDTD (version 8.15.736), a commercial finite-difference time-domain solver. The substrate is semi-infinite in the *z* axis and infinite in the *x*/*y* axis. The size of NP is set as 60 nm in diameter. The refractive index parameter of metal, gold, and silver specifically are support by CRC [46]. Total-field scattered-field source (TFSF), a special designing light source for studying particle scattering, is adopted in our research. The light normally incident from + *z* direction (designed as front incident) and − z direction (designed as back incident). Perfectly matched layers (PMLs) were used to absorb the scattered radiation in all directions (in order to eliminate reflection back to the model). The PML parameters such as Kappa, Sigma, layers, and polynomial order are assumed by 2, 1, 32, and 3 respectively. In addition, FDTD method consists in introducing a space and time mesh that must satisfy the stability criterion [47]. In order to converge, the simulation time and time steps (dt) are set to 2000 fs and 0.07 fs respectively. The space mesh is set to 0.3 nm in every direction (*dx* = *dy* = *dz*).

## Abbreviations

FDTD: Finite-difference time-domain
LSP: Localized surface plasmon
NPs: Nanoparticles
SERS: Surface-enhanced Raman scattering
TERS: Tip-enhanced Raman scattering
TFSF: Total-field scattered-field
